# The role of AMPA receptors in postsynaptic mechanisms of synaptic plasticity

**DOI:** 10.3389/fncel.2014.00401

**Published:** 2014-11-27

**Authors:** Thomas E. Chater, Yukiko Goda

**Affiliations:** RIKEN, Brain Science InstituteWako-shi, Japan

**Keywords:** AMPAR, homeostatic plasticity, Hebbian plasticity, synaptic plasticity, synaptic transmission, trafficking

## Abstract

In the mammalian central nervous system, excitatory glutamatergic synapses harness neurotransmission that is mediated by ion flow through α-amino-3-hydroxy-5-methyl-4-isoxazolepropionic acid receptors (AMPARs). AMPARs, which are enriched in the postsynaptic membrane on dendritic spines, are highly dynamic, and shuttle in and out of synapses in an activity-dependent manner. Changes in their number, subunit composition, phosphorylation state, and accessory proteins can all regulate AMPARs and thus modify synaptic strength and support cellular forms of learning. Furthermore, dysregulation of AMPAR plasticity has been implicated in various pathological states and has important consequences for mental health. Here we focus on the mechanisms that control AMPAR plasticity, drawing particularly from the extensive studies on hippocampal synapses, and highlight recent advances in the field along with considerations for future directions.

## Introduction

The birth of modern neuroscience arguably started with the seminal work of Cajal (1852–1934, Doyle, 1939) who identified neurons as individual units embedded within the vastly complex network of brain tissue. However, little was known about how these intricate and beautiful cells communicated with each other until the advent of more sophisticated techniques that allowed probing of the communication across the synaptic cleft. Studies at the neuromuscular junction, an experimental preparation that was more accessible than the brain, demonstrated that postsynaptic receptors were largely stable and were generally unresponsive to changes in activity level (Fambrough and Hartzell, 1972; Sanes and Lichtman, 1999). Whether this applied to the central nervous system was begun to be answered in the 1970s and 80s, when Bliss and Lømo, working in rabbit hippocampus, first demonstrated that a stimulus could cause an increase in synaptic strength that was long lasting, termed long-term potentiation (LTP: Bliss and Lømo, 1973). The discovery of LTP set in motion the background for the flurry of studies aimed to test if memories are stored at subsets of synapses distributed throughout neuronal networks, and if changes in these tiny structures underlie the ability to learn new behaviors. A particular class of glutamatergic receptors, the α-amino-3-hydroxy-5-methyl-4-isoxazolepropionic acid receptors (AMPARs: Beneyto and Meador-Woodruff, 2004), is a key determinant of synaptic strength, and the plasticity of AMPARs is the focus of this review. This is a large field that has spanned over three decades now, and its progress has relied on diverse experimental approaches using *in vitro* and *in vivo* preparations, from biochemistry, cell biology, electrophysiology, to state-of-the-art imaging combined with increasingly sophisticated genetic manipulation.

After a brief introduction to the discovery and history of AMPARs, this review focuses on their role in postsynaptic plasticity in the hippocampus and the recent advances over the last few years. How do AMPARs initially get to the cell surface, and once there, how are they targeted to and retained at synapses? Neighboring synapses sharing the same dendrite may experience significantly different activity levels, and this impacts AMPAR mobility and synaptic retention. Furthermore, AMPAR subunits are differentially regulated by neuronal activity, especially with respect to enzyme-mediated phosphorylation/dephosphorylation cycles that drive their insertion or removal from the synapse. The incorporation of calcium-permeable AMPARs into synapses in response to stimuli is also an important modulation. Neurons are capable of a variety of plastic changes, and synapse strength is both regulated locally and across thousands of synapses cell-wide. How are AMPARs differentially regulated by these separate forms of plasticity? Finally we will discuss changes in AMPAR plasticity in age-related cognitive decline and brain pathologies, and the implications for normal neuronal function.

## What are AMPARs?

AMPARs are tetrameric, cation-permeable ionotropic glutamate receptors, and are expressed throughout the brain (Beneyto and Meador-Woodruff, 2004). The four AMPAR subunits (GluA1–GluA4) are encoded by the genes GRIA1-GRIA4, and are assembled as dimers-of-dimers to form the hetero-tetrameric receptors (Hollmann and Heinemann, 1994; Traynelis et al., 2010), although homo-tetrameric receptors have been reported (Wenthold et al., 1996; Lu et al., 2009). Upon binding of glutamate, the pore opening allows the influx of Na^+^ ions (along with K^+^ efflux) to depolarize the postsynaptic compartment; however, depending on the subunit composition and the RNA editing, AMPARs also permit Ca^2+^-influx, which has important consequences for plasticity by engaging Ca^2+^-dependent signaling events.

The four AMPAR subunits are highly homologous (around 70% amino acid residue identity) with conserved transmembrane and extracellular domains (Collingridge et al., 2004). The C-terminal intracellular tails are diverse amongst the subunits, and alternative splicing and RNA editing contribute to additional variants. Alternative splicing at the so-called flip/flop exon produces subunit variants with distinct receptor desensitization properties (Lambolez et al., 1996). Moreover, in the adult brain, most GluA2 subunits undergo RNA editing that replaces a glutamine with a positively charged arginine in the pore-forming region of the assembled channel; this Q/R editing prevents Ca^2+^-influx. Therefore, in the adult brain, the majority of GluA2-containing AMPARs are largely Ca^2+^-impermeable (99%, Greger et al., 2003) and they also show a lower single channel conductance (Cull-Candy et al., 2006; Traynelis et al., 2010) along with a slightly increased decay time. In contrast, GluA2-lacking AMPARs are Ca^2+^-permeable (CP-AMPARs), and have a higher single channel conductance (Swanson et al., 1997) and faster rise and decay kinetics. GluA2-lacking AMPARs also display an intracellular block by polyamines, which can be displaced by stimuli delivered close to one another; this phenomenon manifests as a postsynaptic form of paired-pulse facilitation of synaptic responses (Rozov and Burnashev, 1999). A precise role for CP-AMPARs in synaptic plasticity is hotly debated (see below).

## Where are AMPARs located?

AMPARs are enriched at excitatory glutamatergic synapses, where they sit in the postsynaptic membrane opposite the presynaptic active zone where glutamate-filled vesicles fuse with the plasma membrane and release their contents into the synaptic cleft. The number of AMPARs at a particular synapse ranges from tens to hundreds, and at mature synapses, it correlates well with spine size and synaptic strength (Matsuzaki et al., 2001). AMPARs are highly dynamic, showing lateral mobility along the cell surface between synaptic and extrasynaptic regions and also undergo constitutive trafficking to and from the cell surface with a surface half-life measured in tens of minutes (Nishimune et al., 1998). Changes in AMPAR number at the synapse is one of the major ways by which the efficacy of synaptic transmission can be altered. Following patterned neuronal activity, AMPARs shuttle into or out of synapses, resulting in long lasting changes in synaptic strength (Lüscher et al., 1999). LTP and long-term depression (LTD) are the most actively studied forms of synaptic plasticity that are thought to represent cellular correlates of particular types of learning and memory.

Prior to reaching synapses, AMPAR trafficking from the endoplasmic reticulum is regulated by various accessory proteins (for example TARPs and cornichons, see Haering et al., 2014) and deficits in these proteins lead to dysregulation in AMPAR trafficking and their expression at synapses. Along dendrites, AMPARs are trafficked through interactions with kinesin (Perestenko and Henley, 2003; Shin et al., 2003) and GRIP1 (Setou et al., 2002), although dynein may also play a role (Kapitein et al., 2010). Some AMPARs may be inserted into the plasma membrane at the soma and then laterally diffuse along the cell surface to synapses (Adesnik et al., 2005). Importantly, the mRNA coding for GluA1 and GluA2 AMPAR subunits can be detected in dendrites together with protein translation machinery (Grooms et al., 2006). Accordingly, many studies have demonstrated the occurrence of local dendritic translation of GluA1 and GluA2, and that such events can supply AMPARs in these cellular compartments under basal conditions and in response to changes in neuronal network activity (Steward and Levy, 1982; Tang and Schuman, 2002; Ju et al., 2004; Grooms et al., 2006). As we will see below, synapses and their complement of glutamate receptors are able to be regulated at every level, from a single synapse, to a dendritic branch, and in some cases, across the entire neuronal arbor. How the control mechanisms operating at different subcellular domains interact with each other and are synergistically integrated within a single neuron is an exciting topic of research.

## How do AMPARs arrive at the synapse?—AMPAR insertion at the plasma membrane

**Figure 1:**
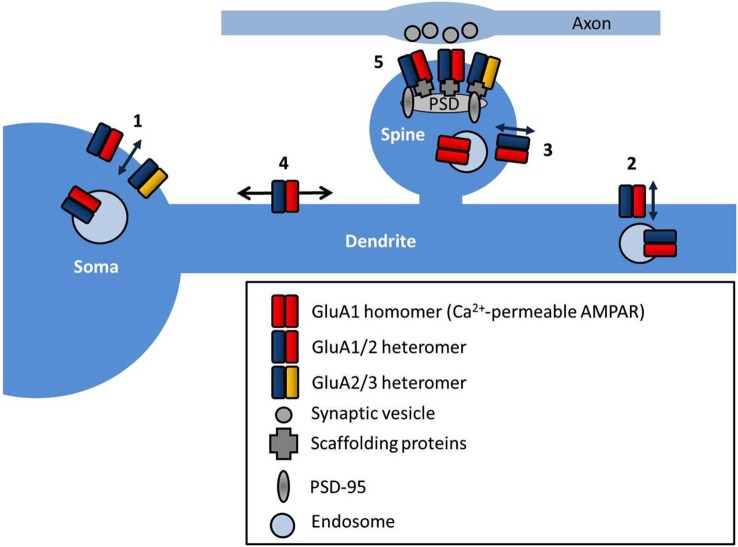
**AMPAR subcellular localization and sites of trafficking**. AMPARs are exocytosed at multiple locations in neurons such as the soma (1), dendrites (2) and directly into the spine (3). AMPARs freely diffuse at the cell surface extrasynaptically (4), and are trapped at synapses by interactions with scaffold proteins at the postsynaptic density (PSD) (5).

The site of exocytosis of AMPARs is not completely clear. Various studies have suggested the insertion site as the soma (Adesnik et al., 2005), dendrite (Yang et al., 2008; Makino and Malinow, 2009; Patterson et al., 2010), or the spine, directly (Wang et al., 2008; Kennedy et al., 2010, see Figure 1). The consensus is that AMPARs are first delivered to extrasynaptic regions, and then diffuse into synapses where they are retained, and both steps are regulated by neuronal activity. AMPAR exocytosis is mediated by SNARE proteins (soluble NSF attachment protein receptors; Lüscher et al., 1999) and synaptic receptors are removed by dynamin-dependent endocytosis (Carroll et al., 1999), although they may be first trafficked laterally along the cell surface away from the synapse. Different AMPAR subunits display distinct exocytosis properties. In general, short-tailed heterodimers (GluA2/3) cycle continuously in and out of the membrane, and maintain the surface pool of synaptic receptors (Passafaro et al., 2001; Shi et al., 2001), whilst AMPARs containing long-tailed subunits (GluA1/2 and GluA2/4) are inserted into synapses in an activity-dependent manner (Hayashi et al., 2000; Shi et al., 2001). Simply increasing the number of extrasynaptic AMPARs is not sufficient to potentiate synapses (for example by overexpression of stargazin, see Schnell et al., 2002), implying that other additional steps are required to stabilize the receptors at the synapse. Postsynaptic density (PSD)-95 appears to fulfill this role, as PSD-95 overexpression selectively promotes synaptic accumulation of AMPAR without altering surface AMPAR number (Bats et al., 2007).

That exocytosis of AMPARs mediates the increase in synaptic strength during LTP in hippocampal CA1 neurons is supported by findings in which blocking dendritic membrane fusion events with botulinum toxins or by infusing peptides that interfere with NSF binding to SNAP, impairs the magnitude of synaptic potentiation (Lledo et al., 1998). Conversely, inhibiting endocytosis or interfering with the interaction between NSF and GluA2 prevents LTD expression (Lüscher et al., 1999), highlighting the importance of AMPAR trafficking in the expression of synaptic plasticity.

Tagging AMPAR subunits extracellularly with the pH-sensitive GFP mutant, super ecliptic pHluorin (SEP, Miesenböck et al., 1998), which is quenched in acidic endosomes but fluoresces brightly at the surface, has facilitated direct monitoring of cell surface AMPARs. Imaging studies using these SEP-tagged AMPAR subunits have provided insights into the temporal relationship between spine structural changes and the delivery of receptors to the synaptic plasma membrane as well as the order of accumulation of different receptor subunits at synapses. Using a chemical LTP (chemLTP) induction protocol in hippocampal organotypic slices, Kopec et al. (2006) have shown that SEP-GluA1 (and to a lesser extent SEP-GluA2) enter spines upon stimulation, and this is preceded by a structural enlargement of the spine head. The timing of subunit insertion that follows the spine enlargement is also supported by electrophysiology experiments using pairing-induced LTP (Hayashi et al., 2000) and by *in vivo* experience-driven forms of plasticity at the barrel cortex (Takahashi et al., 2003) and associative fear conditioning in the lateral amygdala (Rumpel et al., 2005). Another study in cultured hippocampal neurons used total internal reflection microscopy (TIRF) to limit the SEP-AMPAR signals to those very close to the membrane (Tanaka and Hirano, 2012). Careful monitoring of the temporal order of GluA1, GluA2 and GluA3 insertion following LTP-type stimuli has revealed a fast insertion of GluA1 (within 5 min) followed by GluA2 (5–10 min) and finally GluA3 (20–30 min).

Other imaging studies have suggested the existence of multiple types of AMPAR insertion events that are reminiscent of the different modes of synaptic vesicle exocytosis at the presynaptic terminal. Similarly to full collapse vesicle fusion and kiss-and-stay or kiss-and-run fusion events that have been reported for neurotransmitter release, on the postsynaptic side, some AMPAR insertion events involve receptor delivery to the plasma membrane followed by a quick diffusion of the receptors away from the insertion site that is compatible with full collapse, whilst others show retention of the AMPAR clusters at the cell surface for tens of seconds that is similar to the kiss-and-stay mode (Yudowski et al., 2007; Jullié et al., 2014). Whether these different classes of events indicate a difference in cargo function or content is not yet clear, nor whether neuronal activity can bias the delivery mode towards one or the other. However, it seems logical that these variations in the mode of AMPAR insertion are mechanistically linked to the cellular demand for synaptic components. As discussed below, the extrasynaptic pool of AMPARs acts as the source of receptors for synapses to capture. Petrini et al. (2009) showed that after potentiation synaptic AMPAR number increased due to increased receptor exocytosis and stabilization at the synapse. Curiously, disrupting peri-synaptic sites of receptor endocytosis also impaired potentiation, suggesting that constitutively cycling of AMPARs to and from the surface is required for the correct expression of plasticity. Several studies have suggested that the spine neck provides a mechanical intracellular diffusion barrier (Kusters et al., 2013), and that recruitment of AMPARs to the spine can be modified by endocytosis of membrane within the spine (Jaskolski et al., 2009). Clarifying the sites of exo-endocytosis of AMPARs is therefore crucial for understanding the regulation of synaptic strength under basal conditions and in response to synaptic activity.

## “And yet it moves”—AMPAR surface diffusion and plasticity

**Figure 2:**
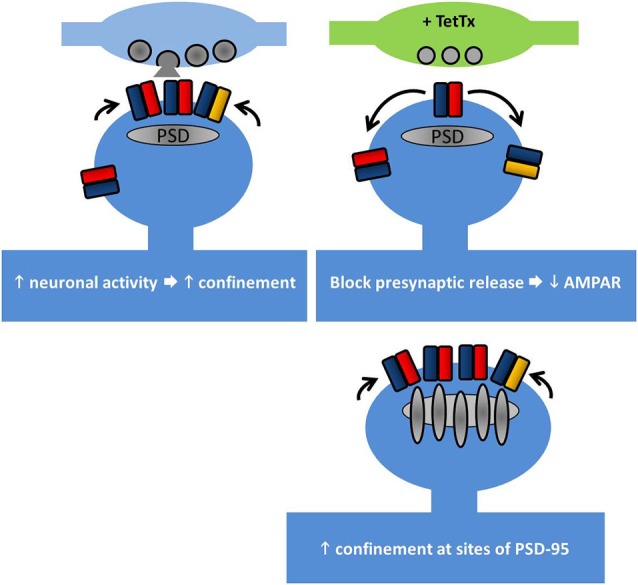
**Lateral diffusion and synaptic retention of AMPARs depends on neuronal activity**. AMPAR retention at synapses is regulated by multiple factors. Top left, increasing neuronal activity reduces the surface diffusion of AMPARs at synapses and increases their trapping. Top right, at individual synapses where the presynaptic release has been chronically blocked, GluA1 retention is reduced. Bottom right, PSD-95 acts to retain AMPARs at synapses. Overexpression of PSD-95 increases synaptic AMPAR accumulation, but not overexpression of stargazin (see main text).

Once at the cell surface, AMPARs are highly mobile and they laterally diffuse along the cell surface. AMPAR diffusion in the plane of the plasma membrane has been mapped using single-particle tracking, showing the contributions of their location, the level of neuronal activity, and the receptor subtype in affecting the type of movement. Whereas extrasynaptic AMPARs diffuse freely, within synapses they exhibit slowing and can become immobilized. In particular, GluA2 subunits diffuse slower in general as neurons mature, and exhibit trapping at synapses. The level of neuronal activity also affects the speed of diffusion, with increased activity slowing the movement of the subunits (Borgdorff and Choquet, 2002; Groc et al., 2004). That slowing of receptor diffusion within synapses could be mediated in part by the interaction with the synaptic scaffold proteins is suggested by the observations in which GluA1 diffusion is slowed at sites of exogenously overexpressed PSD-95, and that GluA1 diffusion is increased upon expressing a stargazin mutant lacking the PDZ-binding motif, which also reduces the immobile fraction of GluA1 (Bats et al., 2007, illustrated in Figure 2).

The role of input activity in controlling receptor diffusion has been elegantly addressed using tetanus toxin (TetTx) to silence individual presynaptic inputs (Ehlers et al., 2007). Postsynapses apposed to TetTx-positive presynaptic boutons tend not to capture GluA1 subunits as they pass through the synapse, despite the slowing of their diffusion (Figure 2). Notably, short-term activity blockade (1–4 h of TTX/APV/CNQX) does not produce the same effect, suggesting that the change in GluA1 diffusion involves a chronic form of structural reorganization at postsynapses lacking presynaptic input activity. Interestingly this study by Ehlers et al. (2007) hints at the existence of nanodomains within the postsynapse (see below) by showing that the confinement radius of AMPARs at active synapses is smaller than at inactive synapses.

The diffusional exchange of AMPARs between synaptic and peri-synaptic regions allows neurons to fine-tune extremely short-term forms of plasticity. AMPARs have a relatively low affinity for glutamate (Lisman and Raghavachari, 2006), and for effective activation they need to be positioned close to or directly opposite presynaptic sites of glutamate release. Cross-linking of surface AMPARs with an antibody to retard their diffusion increases paired-pulse depression (PPD) and decreases the variability of excitatory postsynaptic current (EPSC) amplitude (Heine et al., 2008). This suggests that the rapid diffusional exchange of AMPARs to and from synapses contribute to the recovery from desensitization.

Several other factors can change the synaptic trapping and diffusional properties of AMPARs including corticosteroids (Groc et al., 2008), n-cofillin (Rust et al., 2010), extracellular matrix components (Frischknecht et al., 2009; Szepesi et al., 2014), CaMKII (Opazo et al., 2010) and the endocytosis and recycling of AMPARs (Petrini et al., 2009). It has also been demonstrated that loss of synaptic AMPARs is preceded by transient extrasynaptic endocytosis (Ashby et al., 2004), indicating that the pool of extrasynaptic AMPARs is co-regulated with the synaptic pool. In addition, blocking dynamin to interfere with AMPAR endocytosis can increase AMPAR lateral diffusion (Jaskolski et al., 2009). These observations further support the link between events related to synaptic strength regulation and AMPAR surface motility.

**Figure 3:**
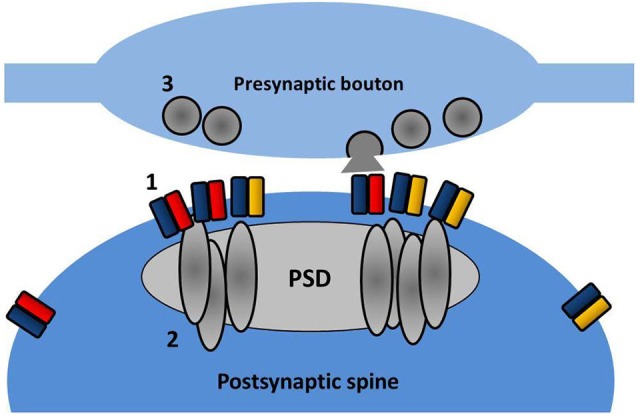
**Nanodomain organization of AMPARs and PSD partners**. Within the spine, AMPARs (1) and PSD-95 (2) are concentrated into sub-diffraction sized clusters. These may reflect the effective positioning of the postsynaptic receptor population opposite presynaptic sites of vesicle fusion (3).

Recently even finer measurements of AMPAR surface diffusion have been made possible with the advent of light-based super-resolution microscopy. Using three different super-resolution approaches (uPAINT, sptPALM, STED), Nair et al. (2013) have revealed the existence of nanodomains (between 60 and 130 nm in diameter) within spine heads where GluA1 and GluA2 subunits are concentrated. Reducing PSD-95 protein levels in neurons decreases the number of receptors per cluster and also reduces miniature EPSC (mEPSC) amplitude, suggesting that these clusters correspond to the postsynaptic target of the presynaptically released glutamate. In a parallel study, a detailed examination of the fine structure of PSD-95 (MacGillavry et al., 2013) has similarly revealed small enriched nanodomains of PSD-95 within the PSD and that these structures can concentrate AMPARs (depicted in Figure 3). The precise functionality of these nanodomains remains to be elucidated, but modeling data suggests that the concentration of AMPARs (and associated scaffold proteins) into nanodomains can strongly affect basal transmission, EPSC variability, and recovery from desensitization (MacGillavry et al., 2013; Nair et al., 2013).

Collectively, these data demonstrate that the level of neuronal activity and modulation of neuronal signaling can control subunit-specific behavior of AMPARs, particularly their incorporation and retention at synaptic sites, and in turn, affect synaptic plasticity. In a simplified model, at synapses, PSD proteins trap and anchor surface AMPARs in response to increases in neuronal or synaptic signaling and release the receptors when activity levels are low. How different forms of activity-dependent synaptic plasticity affect the distribution and composition of synaptic nanodomains is an extremely exciting and promising topic for future research. The postsynaptic nanodomain might be equally matched by the heterogeneous presynaptic organization, for example, representing hotspots of synaptic vesicle priming and fusion.

## LTP—making a memory

**Figure 4:**
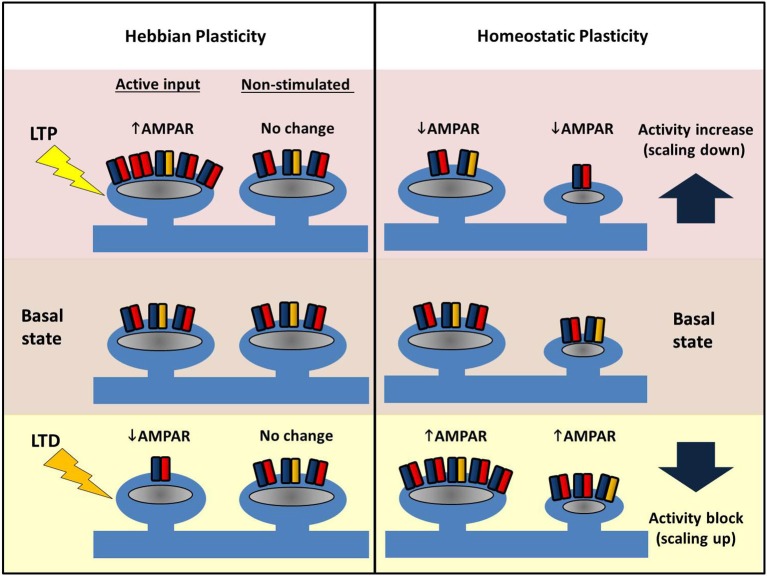
**Comparing Hebbian and homeostatic plasticity**. During Hebbian forms of plasticity synapses change their number of AMPARs in an input-specific fashion. Different patterns of activity can either cause strengthening (LTP, top left) or weakening of synapses (LTD, bottom left) via AMPAR trafficking. Potentiation or depression is limited to stimulated synapses, and neighbors are unaffected. In contrast, during homeostatic plasticity altered levels of neuronal activity drives changes in synaptic AMPAR number across the entire dendritic arbor. Blocking pre- and postsynaptic spiking with TTX causes AMPARs to accumulate at excitatory synapses (bottom right). Conversely increasing network activity (for example with a GABA_A_R antagonist) causes a reduction in synaptic AMPAR (top right). Crucially this form of plasticity conserves the relative strength difference between synapses.

LTP of synaptic strength can be induced by a variety of electrical, pharmacological and behavioral paradigms. Classical LTP, as originally described by Bliss and Lømo (1973) can be stable for months, and presumably mechanisms such as these underlie our own memories, which in humans can span several decades. The key change during LTP is an increase in the number of AMPARs at a subset of synapses (see Figure 4). Presynaptic changes can also contribute to LTP (for reviews, see Kullmann, 2012; Padamsey and Emptage, 2013), but here we focus exclusively on postsynaptic mechanisms.

LTP is typically induced by high frequency tetanic stimulation, which leads to Na^+^-influx through AMPARs, depolarization of the postsynaptic compartment, and activation of NMDARs to permit Ca^2+^-influx; this sets off a cascade of phosphorylation events to potentiate synaptic transmission. The primary change following tetanic stimulation is the gross increase in AMPAR number at the synapse, but hidden within this is a series of subtle temporal and subunit-specific effects. The primary signaling effector (and the most studied molecule) is CaMKII in the postsynaptic neuron. This kinase is transiently activated following LTP induction (Lee et al., 2009), translocates to the synapse (Shen and Meyer, 1999) and phosphorylates target proteins, including GluA1 (Barria et al., 1997; Mammen et al., 1997), whose phosphorylation at S831 enhances single channel conductance (Derkach et al., 1999) and open probability (Banke et al., 2001). Therefore, CaMKII signaling alone can potentiate synaptic transmission, although more recent work suggests that formation of GluA1/2 heterotetramers occludes the S831-mediated increases in channel conductance/open probability (Oh and Derkach, 2005), and places the GluA2 subunit in the dominant role for the secondary modulation of AMPAR function associated with LTP.

In addition to S831, S845 on the GluA1 subunit, which is targeted by PKA, is also found to be phosphorylated after LTP in the hippocampal CA1 region (Barria et al., 1997; Lee et al., 2000). The degree of phosphorylation however depends on the activity history of the synapse (Lee et al., 2000). Knock-in mice that carry at these sites either phosphomimic or phosphonull residues display a lower threshold for spike-timing dependent plasticity and either deficits in LTP or LTD (Lee et al., 2003, 2010; see below for LTD).

PKC is also capable of phosphorylating GluA1, and phosphorylation at S818, which is increased during LTP, is required for LTP induction (Boehm et al., 2006). PKC can also phosphorylate T840, and mutating this site results in deficient LTP in slices prepared from older animals (over 3 months of age) but not from juvenile animals (3–4 weeks old); this suggests an age-dependent component to this form of phosphorylation-dependent modulation of plasticity (Lee et al., 2007).

## LTP and silent synapses

Some synapses have no AMPARs at their resting state and instead just contain NMDARs. Following LTP induction, AMPARs are rapidly trafficked into these “silent synapses” and contribute to the depolarization of the postsynaptic neuron (Isaac et al., 1995; Liao et al., 1995). The existence of silent synapses has been supported by immunolabeling studies in cultured neurons where some synapses only label for NMDARs and not AMPARs (Gomperts et al., 1998; Liao et al., 1999, 2001). The fast “unsilencing” of these synapses during LTP may enable the network to quickly and strongly encode new memories, although more work is needed to clarify how such a form of potentiation could be advantageous over inserting additional AMPARs into existing synapses. Moreover, the detailed molecular basis by which particular silent synapses switch to active ones remains to be established. Presumably alterations in the PSD traps AMPARs at the target synapse, which is paralleled by increased extrasynaptic trafficking of AMPARs to maintain the surface pool.

## Calcium-permeable AMPARs in plasticity

As discussed above, CP-AMPARs, which lack a GluA2 subunit or contain an unedited GluA2 subunit, have a capacity to augment or even replace Ca^2+^-entry through NMDARs to play a role in synaptic plasticity. Exactly how CP-AMPARs contribute to plasticity is unclear, with conflicting evidence in the literature. In one study, LTP induction has been shown to trigger a rapid but transient synaptic insertion of CP-AMPARs that are replaced by GluA2-containing AMPARs within 30 min, and where blocking CP-AMPARs reduces the magnitude of potentiation and CP-AMPARs (Plant et al., 2006). Others have presented data suggesting that CP-AMPARs are delivered to peri-synaptic sites prior to LTP expression (Yang et al., 2008), and that CP-AMPARs maintain the ability of synapses to undergo LTP and spine size expansion (Yang et al., 2010).

Insertion of CP-AMPARs involves phosphorylation events. Guire et al. (2008) showed that CP-AMPAR insertion depends upon CaMKI activity, which in turn requires actin polymerization to recruit synaptic CP-AMPARs, and others have demonstrated a role for PKC phosphorylation (Yang et al., 2010). Another study has linked CP-AMPARs to mEPSC amplitude increases and spine head enlargement following chemLTP in cultured neurons (Fortin et al., 2010), and suggested that downstream of CP-AMPARs, the Rac/PAK/LIM kinase pathway can control spine actin turnover. Phosphorylation of GluA1 at S845 has been reported to play a role in stabilizing GluA1 homomers and retaining CP-AMPARs at peri-synaptic sites (He et al., 2009). The same study has also demonstrated that LTD is accompanied by a reduction of these receptors, and that in mice expressing a GluA1-S845A mutant, peri-synaptic CP-AMPARs are lost. In contrast, no involvement of CP-AMPARs has been seen in hippocampal CA1 LTP in other studies (Adesnik and Nicoll, 2007; Gray et al., 2007).

Multiple studies using GluA2 KO mouse models have demonstrated enhanced LTP in these animals LTP (Jia et al., 1996; Meng et al., 2003; Asrar et al., 2009). Consistently, conditional loss of GluA2 in mice results in increased LTP with no requirement for NMDARs and with no effect on LTD (Wiltgen et al., 2010). These studies also highlight non-overlapping roles of proteins involved in LTP, in that CP-AMPAR-dependent LTP is independent of CaMKII (Asrar et al., 2009) and animals lacking both GluA2 and GluA3 are still able to undergo potentiation (although they show deficits in basal synaptic transmission; Meng et al., 2003).

As discussed below, CP-AMPARs appear to have a role in compensatory, homeostatic forms of plasticity. Perhaps the reported differences in the requirement for CP-AMPARs in LTP and LTD reflect differences in the experimental set up including synapse type, their history of activity, the experimental protocol used to elicit plasticity, and the developmental state of the tissue. The ionic properties of these receptors make them potentially very powerful plasticity players at the synapse. Notably, the mechanism that orchestrates the transient synaptic incorporation of CP-AMPARs is a fascinating one to study. How might some synapses be able to selectively trap GluA2-lacking AMPARs for a short period, only to replace them with GluA2-containing receptors? Does it require a specific set of scaffold proteins with a high binding affinity for GluA2-lacking AMPARs that become unmasked in the PSD?

## LTD—weakening of synapses

Hippocampal synapses are typically bidirectionally plastic, and while LTP may be the cellular correlate of learning and memory, a mechanism to weaken synapses is necessary too. LTD is one such process, and it may underlie forgetting (Nabavi et al., 2014; see Figure 4). Classical hippocampal LTD is dependent on NMDARs (Dudek and Bear, 1992), and its induction engages high affinity Ca^2+^-sensing molecules downstream of the NMDAR activation (Mulkey and Malenka, 1992) such as calcineurin (Mulkey et al., 1994; Jurado et al., 2010). This in turn triggers dephosphorylation events on targets such as GluA1 (Lee et al., 1998, 2000, 2003), leading to depression of synaptic strength via removal of AMPARs (Beattie et al., 2000; Carroll et al., 2001). Although both LTP and LTD are dependent on NMDAR activation and culminate in changes in the number of synaptic AMPARs, the spatio-temporal nature of the intracellular Ca^2+^ rise dramatically impacts the direction of plasticity. GluA1 S845 on the C-terminal tail appears to be required for LTD, as mice carrying an alanine replacement display perturbed LTD (Lee et al., 2010). In contrast, GluA1 S831A mutants show no LTD (or LTP) deficits, whilst the double phosphomutants show impaired LTD as well as a faster decay of LTP (Lee et al., 2003). Interactions between GluA2 and AP2 also contribute to LTD (Lee et al., 2002), and the same region on GluA2 overlaps with the site for NSF interaction, which is required to maintain synaptic AMPAR (Nishimune et al., 1998), but the domain itself is not directly involved in LTD.

A kinase anchoring protein 150 (AKAP150) plays a key role in LTD. AKAP150 can interact with calcineurin and drives NMDAR-dependent removal of AMPARs from the synapse (Jurado et al., 2010). The interplay between AKAP150, PKA and PSD-95 seems particularly important. AKAP150 targets both PKA and PKC to synapses, and the loss of AKAP150 perturbs synaptic transmission (Tunquist et al., 2008). Additionally, preventing PSD-95 interaction with AKAP150 blocks NMDAR-dependent LTD but leaves metabotropic glutamate receptor (mGluR)-LTD intact in cultured neurons (Bhattacharyya et al., 2009). PSD-95 itself undergoes de-phosphorylation at S295 following chemLTD induction (by bath applied NMDA) in cultured neurons, and overexpressing a PSD-95 S295A mutant prevents LTD (Kim et al., 2007).

Another key protein regulating LTD and AMPAR endocytosis is Protein Interacting with C Kinase 1 (PICK1). GluA2 is endocytosed upon phosphorylation at S880 by interacting with PICK1, which also involves PICK1-mediated inhibition of actin polymerization via the Arp2/3 complex (Rocca et al., 2008). This mechanism of AMPAR endocytosis is further regulated by the small GTPase Arf1, and overexpressing a mutant Arf1 that cannot bind PICK1 blocks NMDAR-dependent LTD (Rocca et al., 2013). Additional evidence for the importance of S880 phosphorylation on GluA2 in LTD is provided by the demonstration of increased phosphorylation of this residue following LTD induction (Kim et al., 2001) and of inhibition of LTD upon blocking GluA2/PICK1 interaction (Steinberg et al., 2006). However other groups have shown that S880 phosphorylation of GluA2 can reverse LTD and drive AMPARs to the cell surface by competing with PICK1 binding for GluA2 with GRIP/ABP (Daw et al., 2000). Furthermore, PICK1 knock-down does not prevent NMDA-driven AMPAR removal (Lin and Huganir, 2007). Altogether, these observations point to a role of PICK1 in regulating the intracellular pool of AMPARs after endocytosis, which in turn, can indirectly impact AMPAR internalization.

In the cerebellum there are different forms of LTD; one of the best studied is expressed at synapses between presynaptic parallel fibers and postsynaptic Purkinje cells. This cerebellar parallel fiber LTD shows several key differences compared to hippocampal LTD, including the requirement for GluA2 (Chung et al., 2003), NMDAR-independence (De Zeeuw et al., 1998) and mGluR1 activation (Linden and Connor, 1991). Knocking out GluA2 blocks cerebellar parallel fiber LTD (Chung et al., 2003) as does removing other AMPAR interactors, including PICK1 (Steinberg et al., 2006), and GRIP1 and GRIP2 (Takamiya et al., 2008). Tellingly, reducing endocytosis with inhibitors can block parallel fiber LTD (Wang and Linden, 2000) pointing at a general mechanistic requirement for the removal of AMPARs in LTD regardless of the synapse. Elsewhere in the cerebellum, a form of LTD has been identified at the synapses between mossy fibers and deep cerebellar nuclei. This too is NMDAR-independent, but requires postsynaptic calcium (Zhang and Linden, 2006).

Another well-studied form of LTD crucially involves mGluR activation. Activation of group 1 mGluRs (for example by (R,S)-3,5-dihydroxyphenylglycine, DHPG) induces a rapid removal of synaptic AMPARs. Whilst not covered here, we direct the reader to several excellent review articles on the subject (Gladding et al., 2009; Lüscher and Huber, 2010).

Many other proteins have been shown to modulate LTD to varying extents. Small GTPases Rap1 and Rab5 have both been implicated in hippocampal LTD (Zhu et al., 2002; Brown et al., 2005), along with PI3γ (Kim et al., 2011) and the JAK/STAT signaling pathway (Nicolas et al., 2012). The immediate early gene Arc/Arg3.1 also appears to play a role, as mice lacking this gene have impaired LTD and memory deficits (Plath et al., 2006).

The above notwithstanding, exactly how the behavior of AMPARs determines the outcome of LTD is still unclear, as mice lacking GluA1 (Selcher et al., 2012) or mice lacking both GluA2 and GluA3 (Meng et al., 2003) all show normal hippocampal LTD. In fact, even deleting all four AMPAR subunits and replacing them with kainate receptors can support LTD (Granger and Nicoll, 2014). That LTD generally requires a loss of AMPARs from the synapse seems to be a consistent result. Nevertheless, the exact series of events that drive this loss, and similarly to some aspects of LTP (Granger et al., 2013), the basis for the apparent redundancy of AMPAR subunits remains to be clarified.

## Homeostatic plasticity—non-local and all-encompassing synaptic strength change

In addition to input-specific forms of plasticity, neurons respond to changes in the overall level of network activity, in a cell-autonomous fashion (Maffei and Fontanini, 2009; Vitureira et al., 2012). Individual cells must monitor the level of activity they experience (for example by the state of somatic Ca^2+^-flux following action potentials) and compare it to some pre-set value, and then be able to adjust their synaptic protein complement to offset changes in external activity. In most mammals large changes in network activity happen on a diurnal basis with the onset of sleep (Tononi and Cirelli, 2014), and in pathological states neuronal populations may lose their inputs due to tumorigenesis, focal brain damage or general degenerative syndromes (Small, 2004; Santos et al., 2010). Chronic disease can cripple specific populations of neurons in the brain (for example dopaminergic neurons in Parkinson’s disease) leading to long-term changes in circuit function. This may develop in two stages, with a primary gradual reduction in drive of the affected population, followed by an eventual complete cessation of activity. Other disease states may selectively alter excitatory or inhibitory synapses across the neocortex. These synaptopathies will lead to imbalances across the central nervous system that neurons will attempt to correct as far as their internal mechanisms allow them. Dysregulation of AMPARs at the synapse is the vanguard for many of these diseases, and understanding the mechanisms that counterbalance these perturbations is critical for our understanding of the brain.

Investigations of homeostatic plasticity have often relied on simple, neuronal culture preparations. Experimentally, activity manipulation is achieved in a variety of ways: global pharmacological blockade of synaptic AMPARs and NMDARs heavily suppresses network activity, as does the addition of TTX that prevents action potentials, whereas GABA_A_ receptor blockers increase the overall network activity through disinhibition (Figure 4). In a first demonstration of homeostatic synaptic response monitored by mEPSCs, visual cortical cultures were treated with various channel blockers for 2 days (Turrigiano et al., 1998). Both TTX and AMPAR inhibitor treatment were found to increase the amplitude of mEPSCs whereas bicuculline (a GABA_A_ blocker) decreased the mEPSC amplitude, with the overall effect of maintaining the firing rate of the neuron despite the activity manipulation. Neurons thus adjust their synaptic AMPAR number in a manner that opposes the external changes in activity. Moreover, this is cell-wide and multiplicative such that the differences in individual synaptic weights are conserved. Consequently, this phenomenon—the activity-dependent bidirectional change in mEPSC amplitude—has been termed “synaptic scaling” as all of the individual postsynaptic strengths across the entire neuron are apparently scaled up or down by a uniform amount. Crucially, such a scaling process retains the information encoded in the relative original strengths of the connections, and thus a strong synapse will still be stronger than its weak neighbor after scaling (thus all the work described above on input-specific LTP and LTD is not in vain!). Further studies have indicated that GluA1 and GluA2 increase in a coordinated fashion during scaling up, and AMPA/NMDA ratios are also conserved (Watt et al., 2000). The latter point is interesting to consider with respect to LTP, where in the short-term, the number of AMPARs increases first, and later, NMDARs also increase to restore the ratio (Watt et al., 2004). Similar to LTP and LTD, both scaling up and down of synaptic AMPARs requires Ca^2+^-dependent signaling pathways, some of which are shared (e.g., somatic Ca^2+^-entry), but unlike LTP/LTD, synaptic scaling appears to rely strongly on signaling linked to GluA2 (see below).

Studies have also used local perfusion of drugs to selectively perturb synapses. Interestingly, global action potential firing and local spontaneous mEPSC events appear to play different but overlapping roles in regulating AMPARs. Sutton et al. (2006) has demonstrated that local blockade of NMDARs relieves a brake on local translation to promote the insertion of GluA1. This intriguing finding suggests that individual synapses sense alterations in presynaptic behavior, and are able to respond accordingly. Other strategies to induce local changes in activity have used presynaptic silencing using Kir2.1 (a hyperpolarizing K^+^ channel, which when overexpressed, reduces AP firing, see Burrone et al., 2002) or expressing TetTx to prevent SNARE-dependent neurotransmitter release (Harms et al., 2005), and in both cases synapse-specific responses to the loss of input activity are observed. In addition, local application of TTX onto neuronal somata increases dendritic GluA2 fluorescence within 4 h (Ibata et al., 2008), suggesting that neurons are monitoring their activity level as a function of somatic activity. That this might be somatic Ca^2+^-flux is supported by the finding that blocking all Ca^2+^ channels with NiCl_2_, or L-type Ca^2+^ channels with nifedipine, have the same outcome.

At the level of AMPAR subunits, GluA2 is critical for homeostatic scaling up. Overexpressing a dominant-negative GluA2 C-terminal tail (but not GluA1 C-terminus) blocks this form of plasticity both in cultures and *in vivo* (Gainey et al., 2009). GluA2 KD via siRNA has no effect on basal mESPCs, suggesting that other subunits (largely GluA1) can compensate for the reduced GluA2. GluA2 KD however occluded synaptic scaling but not chemical LTP. As mentioned above, GluA2 KO animals can still express LTP (Jia et al., 1996; Meng et al., 2003; Asrar et al., 2009), and thus altogether these observations hint at non-overlapping functions for GluA2 in different forms of plasticity. A recent study in organotypic hippocampal slices by Arendt et al. (2013) first induced synaptic scaling with TTX and then induced LTP by electrical stimulation. They find that previous activity blockade enhances the subsequent LTP, which appears to be due to the formation of more silent synapses during the activity blockade that are then unsilenced during LTP induction. This suggests that larger structural changes that are not readily discernable may be associated with synaptic scaling.

As of now, multiple proteins have been implicated in synaptic scaling, including Arc (Shepherd et al., 2006), CaMKIV (Ibata et al., 2008), eIF4AIII (Giorgi et al., 2007), retinoic acid (Aoto et al., 2008), Plk2 (Seeburg et al., 2008; Evers et al., 2010), MeCP2 (Blackman et al., 2012), TNF alpha (Stellwagen and Malenka, 2006; Steinmetz and Turrigiano, 2010), beta3 integrins (Cingolani and Goda, 2008; Cingolani et al., 2008), and both PSD-93 and PSD-95 (Sun and Turrigiano, 2011). Beta-catenin KD occludes both scaling up and scaling down and also alters spine shape and decreases mEPSC amplitude without affecting mEPSC frequency. Interestingly, overexpression of a dominant-negative form of N-cadherin mimics the effects of beta-catenin knock down (Okuda et al., 2007; see also Vitureira et al., 2011), supporting the requirement for the N-cadherin/catenin adhesion complex in regulating synaptic AMPARs.

## AMPAR plasticity in disease—where is my mind?

Deficits in synaptic proteins are increasingly implicated in a variety of neurological disorders and neurodegenerative diseases. Any pathological processes affecting the brain will impact synaptic function, although some more directly than others. For example, in Alzheimer’s disease, dysregulated endocytosis of synaptic AMPARs and NMDARs may contribute to progressive memory loss (Tang, 2009). Moreover, amyloid-beta peptide, which is closely linked to Alzheimer’s disease pathology, has been shown to impair synaptic plasticity (Shankar et al., 2008), facilitate hippocampal LTD (Li et al., 2009), and interfere with CaMKII activity and disrupt activity-dependent AMPAR trafficking (Gu et al., 2009). Animal models of Alzheimer’s disease also highlight defects in synaptic AMPAR trafficking and abnormalities in LTP and LTD (Walsh and Selkoe, 2007).

Other disease states or brain syndromes involve alterations in AMPAR subunit composition. Epilepsy causes a loss of GluA1-containing AMPARs across the brain (Grigorenko et al., 1997), whilst exposure to cocaine drives increased levels of CP-AMPARs in dopaminergic neurons in the ventral tegmental area (VTA: Argilli et al., 2008; Bowers et al., 2010; Mameli et al., 2011). In particular, for the latter effect with cocaine exposure, a single dose delivered to a naïve animal produces changes in the VTA that mimics LTP (Ungless et al., 2001; Argilli et al., 2008). Within 3 h, CP-AMPAR expression increases, and renders such synapses unable to undergo a spike-timing-dependent form of LTP. The same VTA response follows injections of morphine, nicotine, ethanol or amphetamine (Saal et al., 2003). Whilst these drug effects are alarming, they at least indicate a potential target for treatment of addiction. Fascinatingly, voluntary administration of cocaine produces a potentiation of these synapses that lasts up to 3 months without further drug use (Chen et al., 2008), as opposed to less than 10 days following a single injection (Ungless et al., 2001).

## Where do we go from here? What are the open questions in AMPAR plasticity?

Undoubtedly, the list of proteins able to regulate synaptic AMPAR levels and their activity remains incomplete. A recent study on AMPAR auxiliary subunits in hippocampal dentate granule cells (DG-GCs) underscores the subtleties still being elucidated (Khodosevich et al., 2014). TARP-γ8 and CKAMP44 are both highly expressed in DG-GCs where they promote AMPAR surface expression and decrease the rate of receptor deactivation. However these two auxiliary subunits have opposite effects on AMPAR desensitization, leading to distinct short-term plasticity; furthermore, only TARP-γ8 is required for LTP expression. This study not only highlights how AMPAR behavior can be uniquely shaped by the cell-type specific expression of modulators with which they complex, but also emphasizes the diversity and flexible control of AMPAR function across the brain.

Whereas AMPAR auxiliary proteins undeniably expand the variety of AMPAR function, in what way do AMPARs with distinct subunit composition contribute to their functional diversity? As discussed above, GluA2-lacking CP-AMPARs participate in different forms of plasticity. Intriguingly, however, a recent report has raised questions about the subunit-specific requirements for recruiting AMPARs to synapses during LTP (Granger et al., 2013). By taking advantage of conditional mice mutants carrying floxed alleles of genes encoding for GluA1, GluA2 and GluA3, Granger et al. tested the effects of genetically ablating any combination of these three subunits on LTP. Surprisingly, they find that any one of the GluA subunits is sufficient for maintaining the enhanced synaptic strength, and even overexpressed kainate receptors can restore LTP in these animals. Using the same approach they have shown that LTD expression is also independent of glutamate receptor subtype (Granger and Nicoll, 2014). Altogether this data suggests that the extra-synaptic surface population of AMPARs is the key factor for providing synaptic receptors for LTP and LTD, although this may depend on the type of stimulus delivered. The unexpected degree of subunit redundancy is remarkable, and even more so given the presence of different accessory proteins that interact with specific glutamatergic receptors to confer the differences in receptor properties. Perhaps experimentally induced LTP is an extreme case of plasticity with reduced discrimination, and under physiological conditions, various aspects of cognitive functions could be driven by controlling and deciphering the subtle variations in synaptic AMPARs. Moreover, changes in AMPAR number, amongst other processes, across a widely distributed set of synapses contribute to network function that ultimately guides behavior, memories, and consciousness. Indeed, how close can we come to physiological stimuli that are sufficient to encode behaviors, whilst we watch the formation of necessary neuronal traces or engrams? This lofty goal may require more than simply changes in AMPARs in synapse remodeling, or the formation of new synaptic and neuronal connections, but would be a genuine high point in our scientific endeavors.

Live super-resolution light microscopy has only just begun to reveal the intricacies of molecular movement at the synapse. Whilst not quite reaching electron microscopy levels of resolution, the advantage of being able to image live tissues at resolutions well below 100 nm makes the technique highly attractive for studying the behavior of synaptic proteins in response to activity. Where exactly do AMPARs undergo exo-endocytic recycling and is this dependent on the subunit composition? Are the nanodomains described for AMPARs and PSD components mirrored by the organization of presynaptic structures? At the active zone, precisely where does presynaptic vesicle fusion take place and in what manner does the released glutamate affect the diffusion properties of synaptic and extra-synaptic AMPARs? Furthermore, targeting super-resolution imaging to *in vivo* synapses in their native milieu, especially in behaving animals, will likely uncover new aspects of AMPAR plasticity that may have been lost in *in vitro* preparations.

In another direction, different disease states are now being unraveled, with the causative genes and protein products being identified in humans and reassembled in animal models. Many pathological states of the brain feature deficits in synaptic transmission at their core, which have been termed “synaptopathies”, and the aging global population has created a serious social and medical issue that neuroscientists must play their part in solving.

## Conflict of interest statement

The authors declare that the research was conducted in the absence of any commercial or financial relationships that could be construed as a potential conflict of interest.
